# ^18^F-FDG whole-body PET/CT for the evaluation of suspected native valve infective endocarditis

**DOI:** 10.1186/s41824-024-00207-7

**Published:** 2024-07-22

**Authors:** Shihan Chen, Noah Ben-Ezra, Stephan Probst, Gad Abikhzer

**Affiliations:** 1 Faculty of Medicine and Health Sciences, McGill University, 3605 Rue de la Montagne, Montreal, QC H3G 2M1 Canada; 2 Department of Nuclear Medicine, Jewish General Hospital, McGill University, Montreal, Canada

**Keywords:** ^18^F-FDG-PET/CT, Infective endocarditis, Septic pulmonary embolism, Diabetic foot osteomyelitis

## Abstract

^18^F-FDG-PET/CT is indicated in the workup of patients with suspected infective endocarditis to detect intra-cardiac and disseminated infections, as well as its source. We present the case of a 66-year-old female patient known for recurrent diabetic foot infection, with equivocal TTE results and persistent MRSA bacteremia despite medical management. PET/CT revealed evidence of left foot osteomyelitis. Whole body PET/CT diagnosed native mitral valve infective endocarditis (IE) and right lower lobe segmental pulmonary artery uptake, consistent with septic pulmonary embolism (PE).

## Key message

Whole-body FDG-PET/CT can contribute to the diagnosis, source assessment, and embolic detection of NVE.

## Introduction

Infective endocarditis (IE) is defined as infection of the endocardium, heart valves, or indwelling cardiac devices (Cahill et al. 2016). Despite recent advancements in diagnostic and therapeutic strategies, the incidence of IE has been increasing, affecting 3–10 out of 100,000 individuals per year in developed countries (Cahill et al. 2016; Pant et al. 2015). IE is associated with high morbidity and mortality rates, partially due to diagnostic challenges and its cardiac/extra-cardiac complications (Abegaz et al. 2017; Mocchegiani et al. 2009). Recent updates to the Duke Criteria have incorporated ^18^F-FDG-PET/CT findings as a major diagnostic criterion for both prosthetic (PVE) and native valve infective endocarditis (NVE) (Fowler et al. 2023). ^18^F-FDG-PET/CT has significantly lower sensitivity in detecting NVE than PVE, but with higher specificity (Pijl et al. 2021). While proper suppression and technological advances in PET/CT resolution improve sensitivity for the detection of NVE, there remains a scarcity of data on this matter to date (Abikhzer et al. 2022). In this case report, we demonstrate the value of ^18^F-FDG PET/CT in the diagnosis, source assessment and embolic detection of NVE.

## Case presentation

**Fig. 1:**
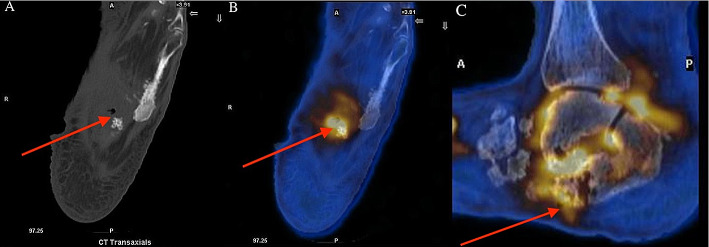
^18^F-FDG-PET/CT of left foot showed intense uptake tracked from a soft tissue ulcer. **A-C**, PET and non-contrast CT performed 95 min after the administration of ^18^F-FDG revealed intense uptake in the soft tissues, bones, and joints of the left hindfoot/midfoot. FDG uptake tracked from a soft tissue ulcer in the plantar surface of the foot (**A-B**, arrow) to the adjacent calcaneus (**C**, arrow)

A 66-year-old female, known for atrial fibrillation, peripheral vascular disease, chronic diabetic foot infection, and recurrent osteomyelitis, presented with unwitnessed fall, fever and Methicillin-resistant Staphylococcus aureus (MRSA) bacteremia. Laboratory tests were notable for elevated WBC count of 23.4 × 10^9^/L (normal range 4.0–11.0 × 10^9^/L) and CRP of 147 mg/L (normal range 0–10 mg/L). She was started on IV vancomycin. ^18^F-FDG-PET/CT (Discovery MI, GE HealthCare) of the feet to rule out diabetic foot osteomyelitis was requested and following a 12 h fast was performed 95 min after injection of ^18^F-FDG with a glycemia of 11.4 mmol/L. ^18^F-FDG-PET/CT showed intense uptake in the bones and joints of the left hindfoot/midfoot, consistent with neuropathic osteoarthropathy. Inferiorly, FDG uptake tracked from a soft tissue ulceration in the plantar surface of the foot to the adjacent calcaneus (Fig. 1). Overall, findings were compatible with neuropathic osteoarthropathy and superimposed osteomyelitis.

**Fig. 2:**
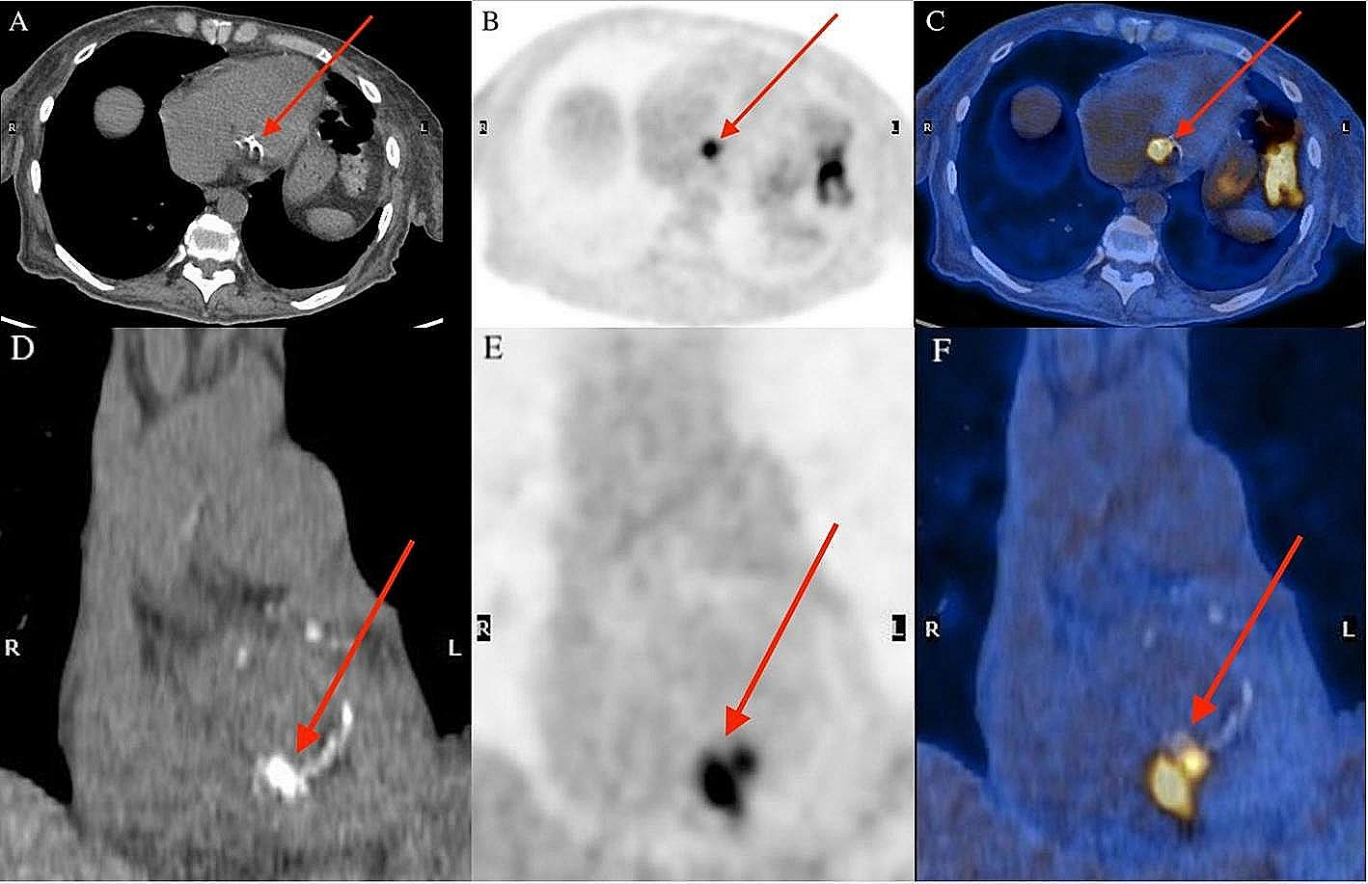
**A-F**, ^18^F-FDG whole-body PET/CT was performed to evaluate for a possible infectious etiology of the mobile right atrium mass visualized on TTE. The study revealed intense, focal FDG uptake (SUVmax 6.1) at the calcified mitral valve, consistent with mitral valve infective endocarditis (**A-F**, arrow). No FDG-avid lesion was observed in the right atrium corresponding to the mobile mass described on TTE.

**Fig. 3:**
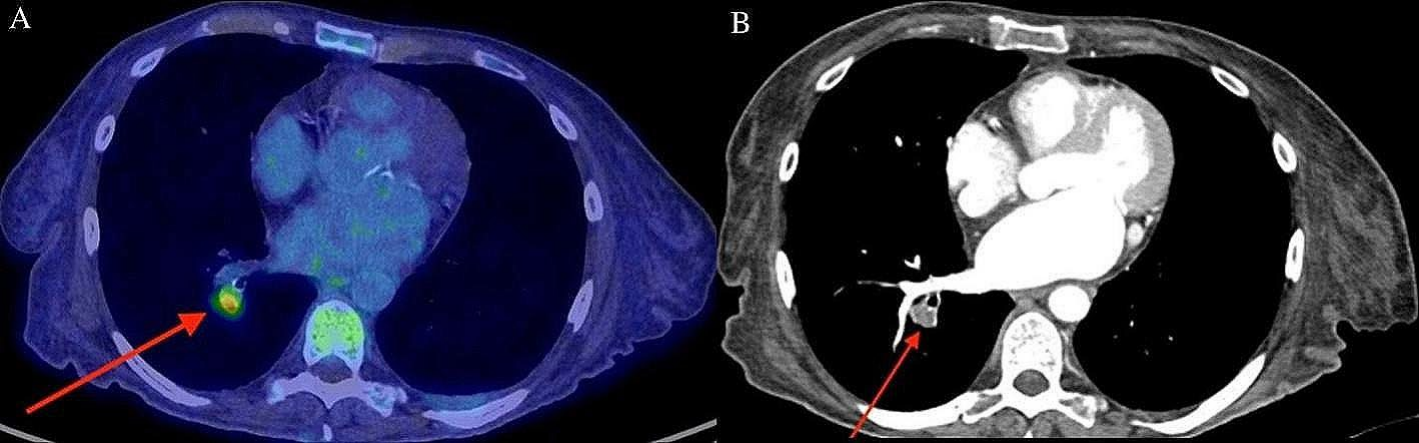
^18^F-FDG whole-body PET/CT and CT pulmonary angiography showed evidence of septic pulmonary embolus in RLL segmental arteries. ^18^F-FDG whole-body PET/CT revealed increased FDG uptake (SUVmax 3.8) in branching of segmental arteries from the right lower lobe (RLL) pulmonary artery, suspicious for septic pulmonary embolus (**A**, arrow). Subsequent CT pulmonary angiography study revealed RLL distal lobar and segmental emboli (**B**, arrow)

Transthoracic echocardiogram (TTE) subsequently revealed a large 21 × 28 mm serpiginous, multilobulated mobile mass in the right atrium without a clear attachment point to a fixed cardiac structure. The differential diagnosis included an atypical vegetation, Chiari network or myxoma. No vegetations were seen on the cardiac valves and there was no evidence of a patent foramen ovale. ^18^F-FDG PET/CT was requested to evaluate for a possible infectious etiology of the right atrial mass. Following a myocardial suppression diet preparation and 18 hour fast, ^18^F-FDG whole-body PET/CT (Discovery MI, GE HealthCare) was performed with glycemia of 4.9 mmol/L. Images from vertex to mid thighs were acquired. The study revealed focal, intense FDG uptake at the calcified mitral valve consistent with mitral valve endocarditis in the clinical context (Fig. 2). Although no FDG-avid lesion was observed in the right atrium, there was increased uptake at the branching of segmental arteries from the right lower lobe (RLL) pulmonary artery, suspicious for septic pulmonary embolus (Fig. 3-A). Subsequent CT pulmonary angiography study revealed RLL distal lobar and segmental emboli, without evident masses in the right atrium (Fig. 3-B). On TTE performed 2 weeks later, the right atrial lesion was no longer present and therefore represented a vegetation in-transit. Daptomycin was added to the treatment regime, and the cultures sterilized afterwards. The source of the MRSA bacteremia and IE was the diabetic foot infection. The patient was deemed not a surgical candidate due to frailty and was managed with prolonged course of antibiotics.

## Discussion

The European Society of Cardiology (ESC) supports the use of ^18^F-FDG-PET/CT as part of the diagnostic work-up of IE (Delgado et al. 2023). Moreover, the 2023 Duke criteria have incorporated ^18^F-FDG-PET/CT findings as a major diagnostic criterion for both prosthetic (PVE) and native valve infective endocarditis (NVE) (Fowler et al. 2023), with nearly perfect specificity for NVE (Kamani et al. 2020). Nevertheless, ^18^F-FDG-PET/CT has a lower sensitivity for detecting NVE compared to that of PVE (Pijl et al. 2021). NVE often affects moving, valvular vegetations, which are difficult to visualize on PET/CT (Pijl et al. 2021). Furthermore, NVE frequently presents with smaller vegetations, which may be challenging to visualize on PET/CT due to limited spatial resolution and partial volume effect (Pijl et al. 2021). Optimization of PET/CT acquisition protocols and myocardial suppression techniques could improve the sensitivity in detecting NVE (Abikhzer et al. 2022), particularly when digital PET devices are used, as in this case.

As a whole-body test, ^18^F-FDG-PET/CT also evaluates for septic emboli in patients with endocarditis. Both non-infectious and septic pulmonary emboli could present with FDG uptake (Pijl et al. 2021; Singh et al. 2016; Flavell et al. 2014). In this case, however, the focal intense FDG uptake in a segmental pulmonary artery and the clinical context was consistent with a septic PE. Including the lower extremities in the field-of-view when imaging patients with bacteremia or endocarditis can potentially be of added value in also detecting septic emboli or the infection source in selected cases.

Our case report highlights the diagnostic value of a single, integrated test, FDG-PET/CT in assessing the source of bacteremia, the diagnosis of NVE, and the detection of septic emboli.

## Data Availability

The datasets used and/or analysed during the current study are available from the corresponding author on reasonable request.

## Abbreviations

CRP: C-reactive protein
IE: Infective endocarditis
PET: Positron emission tomography
FDG: Fluorodeoxyglucose
CT: Computed tomography
NVE: Native valve infective endocarditis
PVE: Prosthetic valve infective endocarditis
MRSA: Methicillin-resistant Staphylococcus aureus
TTE: Transthoracic echocardiogram
IV: Intravenous
RLL: Right lower lobe
PE: Pulmonary embolism
SUV: Standardized uptake value
OPAT: Outpatient parenteral antimicrobial therapy
